# SAG-DTA: Prediction of Drug–Target Affinity Using Self-Attention Graph Network

**DOI:** 10.3390/ijms22168993

**Published:** 2021-08-20

**Authors:** Shugang Zhang, Mingjian Jiang, Shuang Wang, Xiaofeng Wang, Zhiqiang Wei, Zhen Li

**Affiliations:** 1 College of Computer Science and Technology, Ocean University of China, Qingdao 266100, China; zsg@ouc.edu.cn (S.Z.); weizhiqiangouc@163.com (Z.W.); 2 School of Information and Control Engineering, Qingdao University of Technology, Qingdao 266033, China; jiangmingjian@qut.edu.cn; 3 College of Computer Science and Technology, China University of Petroleum (East China), Qingdao 266580, China; 20210006@upc.edu.cn; 4 MindRank AI Ltd., Hangzhou 311113, China; xiaofeng@mindrank.ai; 5 College of Computer Science and Technology, Qingdao University, Qingdao 266071, China

**Keywords:** drug–target affinity, graph neural network, self-attention

## Abstract

The prediction of drug–target affinity (DTA) is a crucial step for drug screening and discovery. In this study, a new graph-based prediction model named SAG-DTA (self-attention graph drug–target affinity) was implemented. Unlike previous graph-based methods, the proposed model utilized self-attention mechanisms on the drug molecular graph to obtain effective representations of drugs for DTA prediction. Features of each atom node in the molecular graph were weighted using an attention score before being aggregated as molecule representation. Various self-attention scoring methods were compared in this study. In addition, two pooing architectures, namely, global and hierarchical architectures, were presented and evaluated on benchmark datasets. Results of comparative experiments on both regression and binary classification tasks showed that SAG-DTA was superior to previous sequence-based or other graph-based methods and exhibited good generalization ability.

## 1. Introduction

Developing a new drug that gains marketing approval is estimated to cost USD 2.6 billion, and the approval rate for drugs entering clinical development is less than 12% [1,2]. Such massive investments and high risks drive scientists to explore novel and more efficient approaches in drug discovery. Under such circumstances, computer-aided drug design methods, especially the recent deep learning-based approaches, have been rapidly developing and have made key contributions to the development of drugs that are in either clinical use or clinical trials. Among the broad range of drug design phases that computational approaches involve, the prediction of drug–target affinity (DTA) is one of the most important steps, as an accurate and efficient DTA prediction algorithm could effectively speed up the process of virtual screening of potential drug molecules, minimizing unnecessary biological and chemical experiments by refining the search space for potential drugs.

Computational approaches for DTA prediction generally comprise two major steps. First, features of drugs or proteins, or representations/descriptors as alternative expressions, are obtained from raw input data by feature extraction methods. Compared to the original input data, the embedded representations are normally more applicable to the subsequent phase and can achieve better performance. The next step, as previously mentioned, is the classification/regression procedure, where the representations act as inputs and the network outputs as either data labels (i.e., active or inactive) or specific values (i.e., the affinity for each drug–target pair). For the feature extraction methods, earlier research represented drugs and proteins based on human experience or skillfully designed mathematical descriptors, i.e., hand-crafted features [3,4]. In this regard, KronRLS uses pairwise kernels that are computed as the Kronecker product of the compound kernel and the protein kernel for the representations [5]. In the SimBoost model, He et al. defined three types of features separately for the drug, target, and the drug–target pair, each of which contained multiple hand-crafted features [6]. These approaches, despite achieving good performance in the DTA prediction task, depend on chemical insights or expert experiences, which, in turn, restrict further optimizations of these models.

With the rapid advancements in deep learning in the last decade, various data-driven methods were proposed for the description of drugs and target proteins [7,8,9,10,11]. These deep learning approaches differ from hand-crafted features, and features can be extracted automatically through deep learning methods and are proved to be more effective. For deep learning approaches specifically in the DTA area, they can be categorized into non-structure-based and structure-based methods. The former learns the representations from sequential data, which are fingerprints of molecule and acid sequences of protein. For example, DeepDTA [12] used only 1D representations of targets and proteins, and convolutional neural networks containing three layers were applied to both acid sequence and drug SMILES to obtain the representations. Similarly, WideDTA [13] also relied only on the 1D representation, but it differed from DeepDTA in which the drug SMILES and protein sequence were represented as words (instead of characters) that correspond to an eight-character sequence and a three-residual sequence, respectively. In addition, the ligand maximum common substructure (LMCS) of drugs and motifs and domains of proteins (PDM) were utilized and formed a four-branch architecture together with the ligand SMILES and protein sequence branches. DeepCPI [14] leveraged techniques from natural language processing to learn low-dimensional feature representations, including latent semantic analysis for drug embedding and Work2vec for protein embedding. On the other hand, the structure-based methods utilized two-dimensional topology (i.e., graph) [15] or three-dimensional structures [16] for representation extraction. As a type of non-Euclidean data, the molecular graph is irregular with variable size, which makes it difficult to apply traditional deep learning methods such as convolutional neural network (CNN) to it. This type of data graph differs from Euclidean structural data, which are not applicable to many basic operations of traditional deep learning methods. In this regard, the graph neural network (GNN) was proposed to handle graph data, and it put no limit on the size of the input graph, thus providing a flexible format to extract in-depth information within the graph [17]. Following this work, a number of variants of the GNN have emerged in recent years, such as the graph convolutional network (GCN) [18], the graph attention network (GAT) [19], and the gated graph neural network (GGNN) [20], and systems based on these GNN variants have demonstrated ground-breaking performance in many relevant application tasks [21]. Focusing on the drug–target prediction tasks, Tsubaki et al. proposed the application of the GNN to DTA (or compound–protein interaction, CPI) prediction, where the compounds were represented as graphs, and, consequently, the 2D structural information could be kept and extracted using the GNN. The *r*-radius subgraphs and *n*-length subsequence were introduced and were proved to be crucial in improving model performance [22]. Similarly, Gao et al. utilized the GNN for drug representation, whereas the protein descriptors were obtained using long short-term memory (LSTM) [23]. GraphDTA also introduced graph representation to take advantage of the 2D structural information of the drug molecular graph [24]. GraphDTA also discarded the CNN in the drug branch, and it used a three-layer GCN as an alternative for drug representation, while keeping the CNN in the protein branch as in DeepDTA. GraphDTA provided better results than those of the baseline 1D approaches, suggesting a prominent role of structural information. Chemical context can also be considered in order to provide additional features other than the molecular graph itself; for example, DeepGS used embedding techniques of Smi2Vec and Prot2Vec to exploit the chemical context within the drug SMILES and amino sequences. This chemical context was then combined with graph-derived features for DTA prediction [25].

The performance of either the 1D or structure-based representation can be enhanced by introducing attention mechanisms. The attention mechanisms allow the network to focus on the most relevant parts of the input and have been proven to be useful for various tasks [19,26]. For instances, AttentionDTA added an additional attention block following the two branches of the drug and protein, and, therefore, the learned features could be further weighted according to the attention score before they were fed into the fully connected classifying layers [27]. Lim et al. proposed a distance-aware attention algorithm that could capture the most relevant intermolecular interactions within the 3D protein–ligand complex. Such attention mechanisms were proved to be effective when applied to DTA prediction tasks with structural information of a complex [16]. Recently, Lee et al. proposed a novel attention structure that introduced self-attention mechanisms for node pooling named self-attention graph pooling (SAGPool), and it achieved state-of-the-art performances in many graph learning tasks [28]. Inspired by this work, we implemented an SAG-DTA network in this study, which adopted a self-attention graph pooling approach to molecular graph representation. Two architectures, namely, global pooling and hierarchical pooling, were implemented and evaluated, with a detailed comparison of the pooling ratio and scoring method for each architecture.

## 2. Materials and Methods

**Figure 1:**
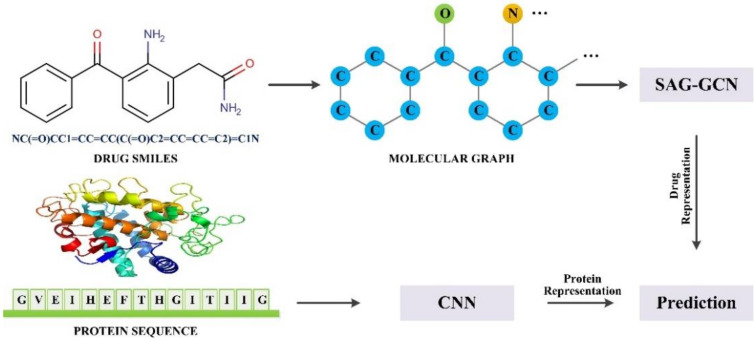
The overall architecture of SAG-DTA.

SAG-DTA is an end-to-end prediction algorithm that takes the SMILES of drug molecules and the acid sequence of proteins as inputs and the affinity value that is measured by either the disassociation constant or KIBA (kinase inhibitors bioactivity data) score as the output. SAG-DTA regards DTA prediction as a regression task, and training data of drug–target pairs are sent to the network, which then learns the intrinsic relationship between the input sample and the output affinity value. Based on the GraphDTA, we implemented a more complicated graph representation of the drug molecule by introducing the self-attention pooling mechanism into the network. Specifically, the atom nodes were weighted by attention scores that were learned based on the features of the nodes themselves. Moreover, the atom nodes were also sorted according to the attention scores, and only those nodes with higher scores were kept. We hypothesized that such modification would allow the network to give more attention to the most important part and thus learn more complex and efficient feature representations for the prediction task. The overall architecture of SAG-DTA is presented in Figure 1. It can be seen that the SMILES of the drug molecule was used to build a molecular graph, and then the graph was sent to the GCN network with SAGPooling layers to learn drug features. For the protein, the acid sequence was sent to the CNN network to learn protein representation.

### 2.1. Datasets

The proposed model was first evaluated on two benchmark datasets of DTA prediction, namely, the Davis [29] and KIBA [30] datasets. The Davis dataset contains selectivity assay data of the kinase protein family and the relevant inhibitors with their respective disassociation constant (*K*_d_) values. The KIBA dataset is about four times the size of the Davis dataset regarding the number of interaction entries. Additionally, it differs from the Davis dataset in that the interaction value was recorded using the KIBA score that was computed from the combination of heterogeneous information sources, i.e., *IC*_50_, *K*_i_, and *K*_d_. The dataset is of high quality, as the integrated heterogeneous measurements mitigated the data inconsistency arising from the use of a single information source. For consistency with previous studies [12,13], the values were transformed into log space (*pK*_d_) using Equation (1).

(1) $$pK_{d}=-\log_{10}\;\left(\frac{K_{d}}{10^{9}}\right)$$

In addition to the DTA prediction datasets, the proposed model was also evaluated on two benchmark binary classification datasets of CPI prediction, namely, the BindingDB [23] and Human [31] datasets. The Human dataset includes positive CPI pairs derived from DrugBank [32] and Matador [33], and it is characterized by the highly credible negative CPI samples. The BindingDB is another well-designed CPI dataset derived from a public database [34], and it contains pre-processed training, validation, and test sets. Statistics of these four datasets are summarized in Table 1.

### 2.2. Input Representation

**Figure 2:**
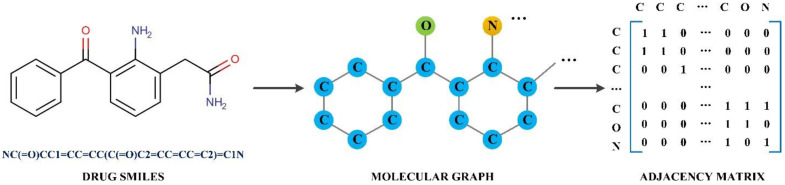
The process of molecular graph construction.

The datasets consist of numerous binding entities, and each entity comprises a drug molecule and target protein pair. The drug molecules were originally stored in the SMILES format, and they were converted to molecular graphs where the atoms and bonds were taken as the nodes and edges, respectively. Self-connection was considered so that the diagonal elements were set to 1. In this study, features for atoms were kept the same as those in GraphDTA and are listed in Table 2. The process was implemented using the RDKit tool (version: 2020.03.4) [35], as shown in Figure 2. For proteins, unique letters that represent categories of amino acids were extracted, and each letter was further represented by integers. The protein sequences could thus be encoded using these integers, which is similar to the method of representation in DeepDTA [12].

### 2.3. Network Architectures

**Figure 3:**
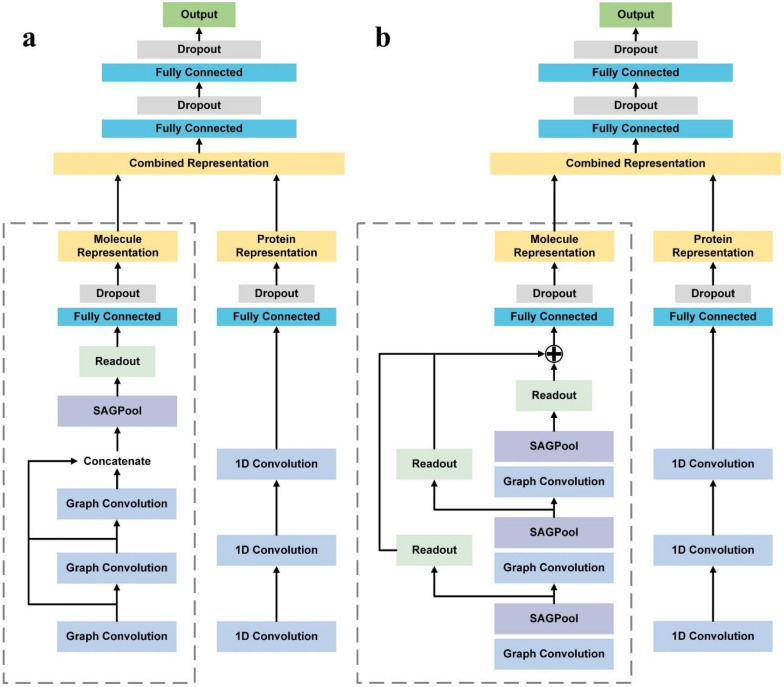
Network architectures of SAG-DTA. Substructures surrounded by dashed lines indicate molecular graph representation, which is the major difference of the two architectures. (**a**) Global pooling architecture. (**b**) Hierarchical pooling architecture.

SAG-DTA network architectures are shown in Figure 3. In this study, we consider two types of architecture in regard to the pooling strategy, namely, the global pooling architecture and hierarchical pooling architecture. The global pooling architecture, as illustrated in the left panel of Figure 3, consists of three graph convolutional layers, and the outputs of these three layers are concatenated before being fed into an SAGPooling layer, i.e., pooling in a global way. The remaining nodes then go through the readout layer and are finally passed to fully connected layers for drug molecule representations. The hierarchical pooing architecture demonstrated in Figure 3b is composed of three blocks, and each of them contains a graph convolutional layer and an SAGPooling layer. The convolutional results of each layer are thus hierarchically pooled and read out. These outputs are then summed before being passed to the fully connected layers to obtain the final drug representations.

#### 2.3.1. Graph Convolution Layer

The graph convolution layer is formulated as Equation (2):

(2) $$h^{\left(l+1\right)}=\sigma ({\tilde{D}}^{-\frac{1}{2}}\tilde{A}{\tilde{D}}^{-\frac{1}{2}}h^{\left(l\right)}\Theta )$$

where $\tilde{A}$ ∈ ℝ*^N^* ^× *N*^ is the graph adjacency matrix with a self-loop, $\tilde{D}$ ∈ ℝ*^N^* ^× *N*^ is the diagonal degree matrix of $\tilde{A}$, *h*^(*l*)^ ∈ ℝ*^N^* ^× *F*^ is the node feature matrix (see Table 2) of the *l*-th layer, and Θ ∈ ℝ*^F^* ^× *F′*^ is the trainable convolution weight with input feature dimension *F* and output feature dimension *F*′. Finally, the rectified linear unit (ReLU) function σ is used as the activation function in our model.

#### 2.3.2. Self-Attention Graph Pooling Layer

**Figure 4:**
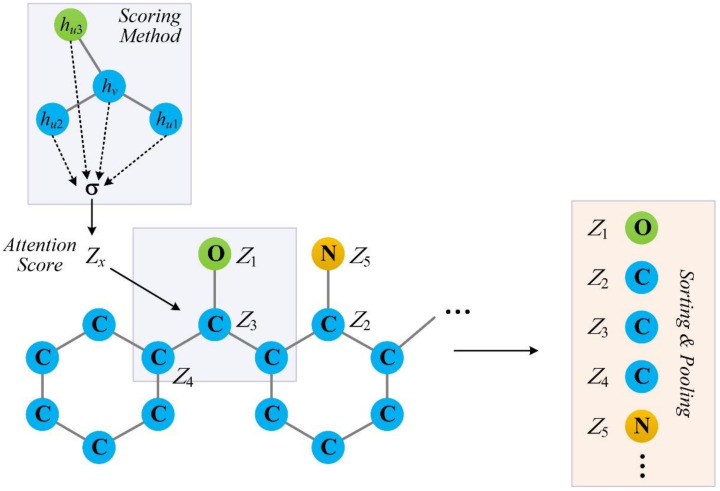
The process of self-attention graph pooling. *V* represents the node itself, and *u*1, *u*2, and *u*3 are the neighbors of node *v*; *h_v_* and *h_u_* are the feature vectors.

The self-attention graph pooling (SAGPool) layer comprise a scoring method and a subsequent mask operation. The process is depicted in Figure 4. Briefly, self-attention scores for all of the atoms in the molecular graph are obtained using certain scoring method; then, all of the nodes are ranked, and the top ⌈*kN*⌉ nodes are selected based on their scores *Z*. *k* ∈ (0,1] is the pooling ratio that indicates the portion of retained nodes.

The mask operation can be formulated as Equation (3):

(3) $$\begin{array}{l} \mathrm{idx}=\mathrm{top}-\mathrm{rank}(Z,\;\lceil kN\rceil \;)\\ Zmask=Z\mathrm{idx} \end{array}$$

where _idx_ is the indexing operation used to obtain the feature attention mask *Z_mask_*.

In this study, four types of scoring methods were evaluated, namely, the GNN, GCN, GAT, and SAGE. These four networks are representative GNN variants and were proved to achieve good performance in graph-related tasks.

##### GNN Scoring Method

The GNN scoring method is defined as Equation (4):

(4) $$Z=\sigma (h_{v}\Theta_{1}+\sum_{u\in N(v)}h_{u}\Theta_{2})$$

where *v* represents the node itself and N(v) is the set of all neighborhoods of node *v*. $h_{v}^{\left(l\right)}$ ∈ ℝ^1^ ^× *F*^ is the feature of node *v* in the *l-*th layer, and $\Theta_{1}$, $\Theta_{2}$ ∈ ℝ*^F^* ^× 1^ are the trainable convolution weights with input feature dimension *F*. σ(⋅) represents the activation function ReLU.

##### GCN Scoring Method

The GCN scoring method is defined as Equation (5):

(5) $$Z=\sigma ({\tilde{D}}^{-\frac{1}{2}}\tilde{A}{\tilde{D}}^{-\frac{1}{2}}h\Theta )$$

Equation (5) is identical to Equation (2), except for the fact that the dimension of convolutional weight is changed to ℝ*^F^* ^× 1^ to obtain the attention score value *Z*.

##### GAT Scoring Method

The GAT scoring method is defined as Equation (6):

(6) $$Z=\sigma ((\alpha_{v,v}h_{v}+\sum_{u\in N(v)}\alpha_{u,v}h_{u})\cdot \Theta )$$

where Θ ∈ ℝ*^F^* ^× 1^ is the trainable convolution weight that is shared by all of the nodes. $\alpha_{v,u}$ is the attention coefficient that is computed as Equation (7):

(7) $$\alpha_{u,v}=\frac{\exp({\mathrm{LeakyReLU}(a}^{T}[\Theta h_{u}||\Theta h_{v}]))}{\sum_{u\in N(v)\cup \{v\}}\exp({\mathrm{LeakyReLU}(a}^{T}[\Theta h_{u}||\Theta h_{v}]))}$$

where ***a*** is the shared attention operation that maps ℝ*^2F′^* to ℝ.

##### SAGE Scoring Method

The SAGE scoring method is defined as Equation (8):

(8) $$Z=\sigma ({\mathrm{mean}}_{u\in N(v)}(\{h_{v}\}\cup \{h_{u}\})\cdot \Theta )$$

where the mean(⋅) indicates an averaging operation.

#### 2.3.3. Readout Layer

The readout layer aggregates node features globally or hierarchically that depend on the pooling architecture. In this work, the readout layer is the concatenation of the average of the max of the node features, which can be written as follows Equation (9):

(9) $$r=\frac{1}{N}\sum_{i=1}^{N}x_{i}∥\overset{N}{\underset{i=1}{\max}}x_{i}$$

where *N* denotes the number of nodes and *x_i_* is the feature vector of the *i*-th node.

## 3. Results and Discussion

The proposed SAG-DTA model contains a number of hyperparameters, and combinations of these hyperparameters form a vast search space. This section presents the evaluation of the two most critical aspects in the self-attention scheme, which are the self-attention pooling ratio and the calculating method for obtaining the attention score. The comparison experiments are detailed in Section 3.3 and Section 3.4. For all of these model evaluation experiments, five-fold cross-validation was used. Specifically, the benchmark training set was shuffled and randomly split into five folds, with four of them being used as the training set and the remainder as the validation set. The model was trained on the four-fold training set and validated on the validation set, and this process was repeated five times. The average result was recorded to assess the model performance. After all of the hyperparameters were determined in this way, we used all five folds to train the model and tested it on the benchmark test set. Finally, we compared SAG-DTA with several existing DTA and CPI prediction methods in Section 3.5 and Section 3.6.

SAG-DTA was implemented using the open-source machine learning framework PyTorch (version:1.4.0) [36] and its extension library PyTorch Geometric (PyG) (version: 1.6.0) [37].

### 3.1. Metrics

In order to make comparisons with the baseline models, the concordance index (CI) and mean squared Error (MSE) were used to evaluate the performances of the model. CI can be used to evaluate the ranking performance of the models that output continuous values [38], and it is computed as Equation (10):

(10) $$CI=\frac{1}{Z}\sum_{\delta_{x}>\delta_{y}}h(b_{x}-b_{y})$$

where *δ_x_* and *δ_y_* are the larger and smaller affinity values, respectively, and *b_x_* and *b_y_* are the corresponding prediction values of the model. *Z* is a normalization constant, and *h*(*x*) is the step function that takes the form of the following Equation [11]:

(11) $$h(x)=\left\{\begin{array}{l} 1,\;\mathrm{if}\;x>0\\ 0.5,\;\mathrm{if}\;x=0\\ 0,\;\mathrm{if}\;x<0 \end{array}\right.$$

The other metric, MSE, measures the difference between the vector of predicted values and the vector of the actual value, and it is widely used in regression tasks. It can be calculated as Equation (12):

(12) $$MSE=\frac{1}{n}\sum_{i=1}^{n}{\left(p_{i}-y_{i}\right)}^{2}$$

where *p_i_* is the predicted value and *y_i_* is the actual value.

The proposed model was also evaluated on several compound–protein interaction (CPI) datasets. CPI prediction is a binary classification task, and the following metrics (Equations (13) and (14)) were used to assess the performance of our models:

(13) $$Presition=\frac{TP}{TP+FP}$$

(14) $$Recall=\frac{TP}{TP+FN}$$

where *TP*, *FP*, and *FN* represent the sample numbers of true positive, false positive, and false negative, respectively.

In addition, the area under the receiver operating characteristic curve (AUROC) and the area under the precision recall curve (AUPRC) of the presented model were also calculated to facilitate comparisons with other models.

### 3.2. Setting of the Hyperparameters

The hyperparameters that were used in SAG-DTA model are listed in Table 3. Most of these hyperparameters were derived from the baseline model (i.e., GraphDTA [24]), while the pooling ratio and the scoring method as two key factors for the performance of SAG were determined in detail using fivefold cross-validation. In this study, we evaluated the performances of these two hyperparameters thoroughly on both the global and hierarchical architectures. The search spaces of the hyperparameters and architectures are highlighted in **bold** in the last three lines of Table 3.

### 3.3. Performances of Various Pooling Ratios

**Figure 5:**
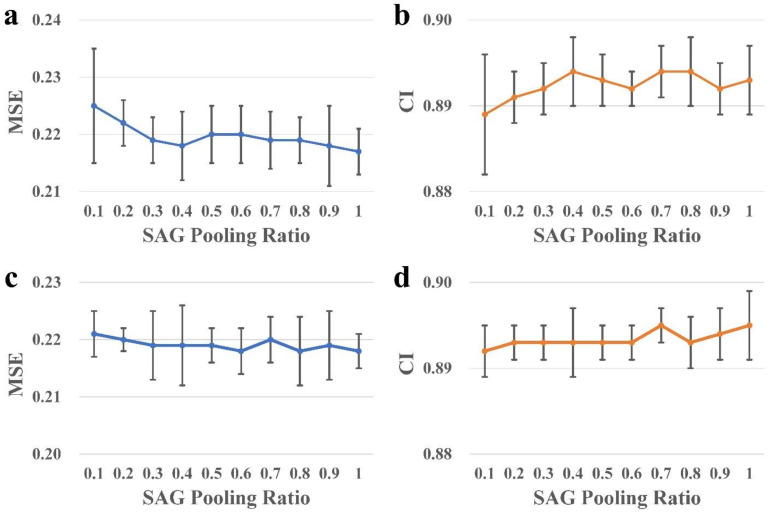
Fivefold cross-validation results when using different pooling ratios. (**a**,**b**) are the MSE and CI results for the global pooling architecture, respectively; (**c**,**d**) are the MSE and CI for the hierarchical pooling architecture, respectively.

The pooling ratio of SAGPool, which determines the percentage of nodes that should be retained, is a key factor to be considered in the model. To identify the best graph pooling ratio, values from 0.1 to 1 were evaluated for both the global and hierarchical pooling architectures, as illustrated in Figure 5.

For the global architecture, the MSE showed a generally downward trend and achieved its lowest value of 0.217 at a pooling ratio 1.0. Another metric CI exhibited oscillation between 0.892 and 0.894 when the pooling ratio was larger than 0.4. The best pooling ratio was finalized as 1.0 in this architecture based on the major indicator MSE.

For the hierarchical architecture, the MSE showed a similar downward trend, with the minimum value of 0.218 achieved at several pooling ratios, including 0.6, 0.8, and 1.0. The ratios were then compared using the candidate CI metric, as demonstrated in the right bottom panel of Figure 5, which is roughly in agreement with the MSE that showed better performance with the increase in the pooling ratio. The ratio value of 1.0 was finally chose, as it achieved the best performance regarding both the MSE (0.218 ± 0.003) and CI (0.895 ± 0.004). The results demonstrate that all atoms in drug molecules had their specific contributions to the drug’s interactions with protein targets. Although assigning weights to nodes could differentiate the contribution of different atoms and therefore benefit the performance of the prediction model, the results suggest that those atoms with lower attention scores cannot be completely ignored.

### 3.4. Performances of Various Attention Scoring Methods

**Figure 6:**
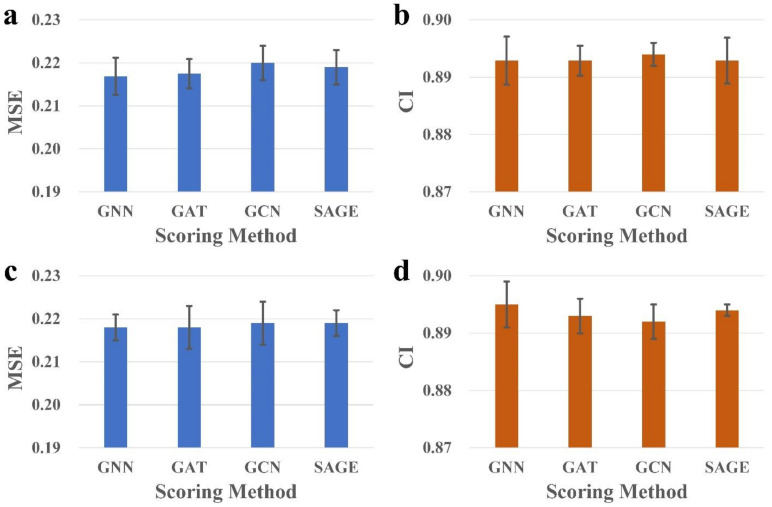
Fivefold cross-validation results when using different scoring methods. (**a**,**b**) are the MSE and CI results for the global pooling architecture, respectively; (**c**,**d**) are the MSE and CI results for the hierarchical pooling architecture, respectively.

The self-attention pooling layer assigns each node an attention score. The attention score has two functions. First, scores of atoms are used as a criterion for ranking and pooling nodes within the graph. Second, the score is used directly as a weighting factor on the node features to differentiate the contribution of different atoms. Since the attention scores directly decide the importance of nodes within each layer, the scoring method thus acts as another important factor in determining the performance of the model and, therefore, needs to be carefully decided. As part of the self-attention pooling strategy, we used the feature of the node itself as the only input feature in the scoring model to obtain the scores of each node, i.e., self-attention. For the scoring method, we adopted the GNN rather than hand-crafted functions to automatically learn the weights. In this section, we compare four GNN variants as scoring methods using fivefold cross-validation, namely, the GNN, GCN, GAT, and SAGE (introduced in Section 2.3.2). The results are illustrated in Figure 6.

For the global architecture, the GNN achieved an MSE of 0.217, which was the lowest among the four scoring methods. The obtained CI values showed slight discrepancies, but the GNN, GAT, and SAGE all achieved a CI value of 0.893. For the hierarchical architecture, the GNN also achieved the best MSE (0.218 ± 0.003) and CI (0.895 ± 0.004). These results together demonstrate that the GNN is the most effective method of the four scoring methods for both the global and hierarchical architectures.

### 3.5. Comparisons with Other Baseline Models

The optimal hierarchical and global SAG models that were obtained via the above hyperparameter tuning were compared to traditional machine learning methods (i.e., KronRLS [5,39] and SimBoost [6]) and recent cutting-edge DTA prediction approaches, including DeepDTA [12], WideDTA [13], AttentionDTA [27], DeepGS [25], and GraphDTA [24]. In these models, different descriptors were used to represent proteins and compounds (the column ‘Proteins and Compounds’ in Table 4), including the Smith–Waterman (S-W) [40] descriptor; the PubChem Sim descriptor [41]; and the descriptors obtained from convolutional networks, such as CNN (for SMILES) and GCN (for graph representation). For WideDTA, the protein sequence (PS) and protein motifs and domains (PDM) were specifically used for protein description, whereas ligand SMILES (LS) and ligand maximum common substructure (LMCS) were used for drug description.

For all of these methods, the same benchmark test sets were used, and the overall performances measured by MSE and CI are summarized in Table 4. It can be seen that SAG-DTA approaches were superior to 1D representation-based approaches or other graph-based approaches. Among the two pooling architectures, the global architecture achieved better performance with an MSE of 0.209 and a CI of 0.903. Though slightly inferior to the global architecture, the hierarchical variant also obtained good results that were better than those of the other baseline models.

To further test the generalization of the proposed method, we evaluated the model on the KIBA dataset with the same hyperparameters as those in the Davis dataset. The experimental results are shown in Table 5, and can be observed that SAG-DTA is the most accurate among the evaluated methods. In detail, the global SAG-DTA achieved an MSE of 0.130 and a CI of 0.892, and the hierarchical SAG-DTA achieved an MSE of 0.131 and a CI of 0.893. These results demonstrate the effectiveness and good generalization ability of our model in DTA prediction.

### 3.6. Model Evaluations of the Compound–Protein Interaction Task

We also assessed the performances of SAG-DTA in CPI prediction. In this study, we refer to the binary classification task of drug–target interaction as CPI to distinguish it from the DTA, which is a regression task. The two architectures of SAG-DTA were separately evaluated on two widely used benchmark datasets of CPI prediction, namely, the Human and BindingDB datasets. These datasets contain compound and protein pairs in addition to a binary label that indicates whether or not they interact. SAG-DTA was slightly adjusted for the binary classification task by adding a Sigmoid layer only in order to ensure that the model was able to predict probabilities and binary labels for samples.

On the Human dataset, SAG-DTA was compared to traditional machine learning algorithms, including k-nearest neighbors (k-NN); random forest (RF); L2-logistic (L2); support vector machines (SVMs); and some recent graph-based approaches, such as CPI-GNN [22], DrugVQA [42], and TransformerCPI [43]. The performances of these models were obtained from [43] and are summarized in Table 6. It can be observed that both SAG-DTA architectures were superior to other methods in terms of AUROC, precision, and recall. Notably, SAG-DTA achieved a significant improvement in the baseline GraphDTA such that the AUROC was improved to 0.984 (±0.003) from 0.960 (±0.005).

The evaluation results on the BindingDB dataset are summarized in Table 7. Among these graph-based methods, SAG-DTA of the global architecture achieved the best performance in terms of AUROC (0.963) and AUPRC (0.966), and the hierarchical architecture variant was also superior to other methods. Notably, hyperparameters of both the two SAG-DTA variants were not fine-tuned for both the Human and BindingDB datasets.

In summary, the superior performance of SAG-DTA on both DTA and CPI tasks suggests its good generalization ability. To provide insight into the improved results by introducing the self-attention algorithm, we discussed the mechanism here in terms of the machine learning aspect as well as chemical intuition.

From a machine learning perspective, the self-attention algorithm in SAG-DTA is a function of the weighting operation that assigns weights, i.e., attention scores, to each atom node within a molecule graph. The features/information of different nodes are therefore weighted before they are aggregated as the final molecule descriptor. Molecular graph descriptors obtained in this way can be more effective, because, in some cases, such as DTA and CPI tasks, the nodes are not equally important for the final prediction. In contrast, for these graph prediction models without self-attention, the node features are indiscriminately aggregated. As a result, the features of some critical nodes are not ‘highlighted’ in the final graph representation.

The above discussions can be naturally extended to the drug molecular graph and DTA/CPI tasks. It can be assumed that atoms in a drug molecule typically do not contribute equally to the final affinity value, and the attention scores can therefore differentiate the importance of different atoms. These critical atoms that play chemical roles in the process of drug–protein interaction will gain more weight when involved into affinity prediction. Consequently, effective representations of molecules are obtained with the help of the self-attention algorithm.

## 4. Conclusions

Predicting drug–target affinity is of great importance to drug development, and an accurate DTA algorithm will benefit the drug screening by minimizing experimental costs and reducing development durations. In this paper, we proposed a graph-based DTA prediction method named SAG-DTA, which utilizes self-attention mechanisms on the drug molecular graph to obtain drug representation. Evaluation of the model on benchmark datasets demonstrated that both hierarchical architecture-based and global architecture-based SAG-DTA achieved superior performance to that of various existing DTA prediction methods, suggesting the effectiveness of the proposed approach in predicting the affinity of drug and protein pairs. Furthermore, the good performance of SAG-CPI, which is the CPI version of SAG-DTA, demonstrated the good generalization ability of the proposed method as well as the effectiveness of the self-attention mechanisms.

## Figures and Tables

**Table 1:** Datasets.

Dataset	Proteins	Compounds	Binding Entities	Task Type	Ref
Davis	442	68	30,056	DTA (regression)	[29]
KIBA	229	2111	118,254	DTA (regression)	[30]
Human	852	1052	3369 (+)/3359 (−)	CPI (binary-class)	[31]
BindingDB	1696	53,253	39,747 (+)/31,218 (−)	CPI (binary-class)	[23]

**Table 2:** Atom features.

Feature	Description	Size
Atom type	C, N, O, S, F, Si, P, Cl, Br, Mg, Na, Ca, Fe, As, Al, I, B, V, K, Tl, Yb, Sb, Sn, Ag, Pd, Co, Se, Ti, Zn, H, Li, Ge, Cu, Au, Ni, Cd, In, Mn, Zr, Cr, Pt, Hg, Pb, or “Unknown” (one-hot)	44
Degree	Number of directly bonded neighbors (one-hot)	11
Num of H	Number of H bound to the atom	11
Valence	Number of implicit H bound to the atom	11
Aromaticity	Whether the atom is aromatic	1
**Total**		**78**

**Table 3:** Hyperparameters used in this study.

Hyperparameters	Setting
Epoch	2000
Batch size	512
Optimizer	Adam
Learning rate	0.001
Dropout rate	0.1
Convolutional layers	3
**SAG pooling architecture**	**global, hierarchical**
**SAG pooling ratio**	**0.1, 0.2, 0.3, 0.4, 0.5, 0.6, 0.7, 0.8, 0.9, 1.0**
**SAG scoring method**	**GNN, GCN, GAT, SAGE**

**Table 4:** Performances of various cutting-edge approaches on the Davis dataset.

Models	Proteins and Compounds	CI	MSE	Ref
KronRLS	S-W and PubChem Sim	0.871	0.379	[5,39]
SimBoost	S-W and PubChem Sim	0.872	0.282	[6]
**1D Representation (SMILES)-Based Approaches**				
DeepDTA	CNN and CNN	0.878	0.261	[12]
WideDTA	PS + PDM and LS + LMCS	0.886	0.262	[13]
AttentionDTA	CNN and CNN	0.887	0.245	[27]
**2D Representation (Graph)-Based Approaches**				
DeepGS	CNN and Graph	0.880	0.252	[25]
GraphDTA(GAT)	CNN and Graph	0.892	0.232	[24]
GraphDTA(GIN)	CNN and Graph	0.893	0.229	[24]
SAG-DTA (HierPool)	CNN and Graph	**0.901**	**0.212**	**Ours**
SAG-DTA (GlobPool)	CNN and Graph	**0.903**	**0.209**	**Ours**

**Table 5:** Performances of various cutting-edge approaches on the KIBA dataset.

Models	Proteins and Compounds	CI	MSE	Ref
KronRLS	S-W and Pubchem Sim	0.782	0.411	[5,39]
SimBoost	S-W and Pubchem Sim	0.836	0.222	[6]
**1D Representation (SMILES)-Based Approaches**				
DeepDTA	CNN and CNN	0.863	0.194	[12]
WideDTA	PS + PDM and LS + LMCS	0.875	0.179	[13]
AttentionDTA	CNN and CNN	0.882	0.162	[27]
**2D Representation (Graph)-Based Approaches**				
DeepGS	CNN and Graph	0.860	0.193	[25]
GraphDTA(GCN)	CNN and Graph	0.889	0.139	[24]
GraphDTA(GAT_GCN)	CNN and Graph	0.891	0.139	[24]
SAG-DTA (HierPool)	CNN and Graph	**0.893**	**0.131**	**Ours**
SAG-DTA (GlobPool)	CNN and Graph	**0.892**	**0.130**	**Ours**

**Table 6:** Performances of various CPI prediction approaches on the Human dataset.

Models	AUROC	AUPRC	Precision	Recall	Ref.
k-NN	0.860		0.927	0.798	[43]
RF	0.940		0.897	0.861	[43]
L2	0.911		0.913	0.867	[43]
SVM	0.910		0.966	0.969	[43]
GraphDTA	0.960 ± 0.005		0.882 ± 0.040	0.912 ± 0.040	[24]
GCN	0.956 ± 0.004		0.862 ± 0.006	0.912 ± 0.010	[18]
CPI-GNN	0.970		0.918	0.923	[22]
DrugVQA	0.964 ± 0.005		0.897 ± 0.004	0.948 ± 0.003	[42]
TransformerCPI	0.973 ± 0.002		0.916 ± 0.006	0.925 ± 0.006	[43]
SAG-DTA (HierPool)	**0.985 ± 0.002**	**0.986 ± 0.002**	**0.945 ± 0.014**	**0.933 ± 0.011**	**Ours**
SAG-DTA (GlobPool)	**0.984 ± 0.003**	**0.984 ± 0.003**	**0.946 ± 0.009**	**0.931 ± 0.014**	**Ours**

**Table 7:** Performances of various CPI prediction approaches on the BindingDB dataset.

Models	AUROC	AUPRC	Precision	Recall	Ref.
CPI-GNN	0.603	0.543			[22]
GCN	0.927	0.913			[18]
GraphDTA	0.929	0.917			[24]
TransformerCPI	0.951	0.949			[43]
SAG-DTA (HierPool)	**0.954**	**0.950**	**0.849**	**0.942**	**Ours**
SAG-DTA (GlobPool)	**0.963**	**0.966**	**0.900**	**0.882**	**Ours**

## Data Availability

All the relevant data are included within the paper.

## Abbreviations

AUPRC	area under the precision recall curve
AUROC	area under the operating characteristic curve
CI	concordance index
CNN	convolutional neural network
CPI	compound–protein interaction
DTA	drug–target affinity
GAT	graph attention network
GCN	graph convolutional network
GGNN	gated graph neural network
KIBA	kinase inhibitor bioactivity data
k-NN	k-nearest neighbors
L2	L2-logistic
LMCS	ligand maximum common substructure
LMCS	ligand maximum common substructure
LS	ligand SMILES
LSTM	long short-term memory
MSE	mean squared error
PDM	motifs and domains of proteins
PS	protein sequence
PyG	PyTorch Geometric
ReLU	rectified linear unit
RF	random forest
SAG	self-attention graph
SAGPool	self-attention graph pooling
SMILES	simplified molecular input line entry specification
SVM	support vector machines
S-W	Smith–Waterman

## References

[1] DiMasi J.A., Grabowski H.G., Hansen R.W. (2016). Innovation in the pharmaceutical industry: New estimates of R&D costs. J. Health Econ..

[2] Mullard A. (2014). New drugs cost US $2.6 billion to develop. Nat. Rev. Drug Discov..

[3] Van Laarhoven T., Marchiori E. (2013). Predicting drug-target interactions for new drug compounds using a weighted nearest neighbor profile. PLoS ONE.

[4] Ding Y., Tang J., Guo F. (2016). Identification of protein--protein interactions via a novel matrix-based sequence representation model with amino acid contact information. Int. J. Mol. Sci..

[5] Cichonska A., Ravikumar B., Parri E., Timonen S., Pahikkala T., Airola A., Wennerberg K., Rousu J., Aittokallio T. (2017). Computational-experimental approach to drug-target interaction mapping: A case study on kinase inhibitors. PLoS Comput. Biol..

[6] He T., Heidemeyer M., Ban F., Cherkasov A., Ester M. (2017). SimBoost: A read-across approach for predicting drug--target binding affinities using gradient boosting machines. J. Cheminform..

[7] Abbasi K., Razzaghi P., Poso A., Ghanbari-Ara S., Masoudi-Nejad A. (2020). Deep Learning in Drug Target Interaction Prediction: Current and Future Perspective. Curr. Med. Chem..

[8] Cherkasov A., Muratov E.N., Fourches D., Varnek A., Baskin I.I., Cronin M., Dearden J., Gramatica P., Martin Y.C., Todeschini R. (2014). QSAR modeling: Where have you been? Where are you going to?. J. Med. Chem..

[9] Zhang S., Golbraikh A., Tropsha A. (2006). Development of Quantitative Structure- Binding Affinity Relationship Models Based on Novel Geometrical Chemical Descriptors of the Protein- Ligand Interfaces. J. Med. Chem..

[10] Politi R., Rusyn I., Tropsha A. (2014). Prediction of binding affinity and efficacy of thyroid hormone receptor ligands using QSAR and structure-based modeling methods. Toxicol. Appl. Pharmacol..

[11] Wang S., Jiang M., Zhang S., Wang X., Yuan Q., Wei Z., Li Z. (2021). MCN-CPI: Multiscale Convolutional Network for Compound--Protein Interaction Prediction. Biomolecules.

[12] Öztürk H., Özgür A., Ozkirimli E. (2018). DeepDTA: Deep drug--target binding affinity prediction. Bioinformatics.

[13] Öztürk H., Ozkirimli E., Özgür A. (2019). WideDTA: Prediction of drug-target binding affinity. arXiv.

[14] Wan F., Zhu Y., Hu H., Dai A., Cai X., Chen L., Gong H., Xia T., Yang D., Wang M.-W. (2019). DeepCPI: A deep learning-based framework for large-scale in silico drug screening. Genom. Proteom. Bioinform..

[15] Zhao T., Hu Y., Valsdottir L.R., Zang T., Peng J. (2020). Identifying drug--target interactions based on graph convolutional network and deep neural network. Brief. Bioinform..

[16] Lim J., Ryu S., Park K., Choe Y.J., Ham J., Kim W.Y. (2019). Predicting drug--target interaction using a novel graph neural network with 3D structure-embedded graph representation. J. Chem. Inf. Model..

[17] Scarselli F., Gori M., Tsoi A.C., Hagenbuchner M., Monfardini G. (2008). The graph neural network model. IEEE Trans. Neural Netw..

[18] Kipf T.N., Welling M. (2016). Semi-supervised classification with graph convolutional networks. arXiv.

[19] Veličković P., Cucurull G., Casanova A., Romero A., Lio P., Bengio Y. (2017). Graph attention networks. arXiv.

[20] Li Y., Tarlow D., Brockschmidt M., Zemel R. (2015). Gated graph sequence neural networks. arXiv.

[21] Zhou J., Cui G., Zhang Z., Yang C., Liu Z., Wang L., Li C., Sun M. (2018). Graph neural networks: A review of methods and applications. arXiv.

[22] Tsubaki M., Tomii K., Sese J. (2019). Compound-protein interaction prediction with end-to-end learning of neural networks for graphs and sequences. Bioinformatics.

[23] Gao K.Y., Fokoue A., Luo H., Iyengar A., Dey S., Zhang P. Interpretable Drug Target Prediction Using Deep Neural Representation. Proceedings of the IJCAI.

[24] Nguyen T., Le H., Venkatesh S. (2019). GraphDTA: Prediction of drug--target binding affinity using graph convolutional networks. BioRxiv.

[25] Lin X. (2020). DeepGS: Deep representation learning of graphs and sequences for drug-target binding affinity prediction. arXiv.

[26] Xiong Z., Wang D., Liu X., Zhong F., Wan X., Li X., Li Z., Luo X., Chen K., Jiang H. (2019). Pushing the boundaries of molecular representation for drug discovery with the graph attention mechanism. J. Med. Chem..

[27] Zhao Q., Xiao F., Yang M., Li Y., Wang J. Attention DTA: Prediction of drug-target binding affinity using attention model. Proceedings of the 2019 IEEE International Conference on Bioinformatics and Biomedicine (BIBM).

[28] Lee J., Lee I., Kang J. Self-attention graph pooling. Proceedings of the International Conference on Machine Learning.

[29] Davis M.I., Hunt J.P., Herrgard S., Ciceri P., Wodicka L.M., Pallares G., Hocker M., Treiber D.K., Zarrinkar P.P. (2011). Comprehensive analysis of kinase inhibitor selectivity. Nat. Biotechnol..

[30] Tang J., Szwajda A., Shakyawar S., Xu T., Hintsanen P., Wennerberg K., Aittokallio T. (2014). Making sense of large-scale kinase inhibitor bioactivity data sets: A comparative and integrative analysis. J. Chem. Inf. Model..

[31] Liu H., Sun J., Guan J., Zheng J., Zhou S. (2015). Improving compound--protein interaction prediction by building up highly credible negative samples. Bioinformatics.

[32] Wishart D.S., Knox C., Guo A.C., Cheng D., Shrivastava S., Tzur D., Gautam B., Hassanali M. (2008). DrugBank: A knowledgebase for drugs, drug actions and drug targets. Nucleic Acids Res..

[33] Günther S., Kuhn M., Dunkel M., Campillos M., Senger C., Petsalaki E., Ahmed J., Urdiales E.G., Gewiess A., Jensen L.J. (2007). SuperTarget and Matador: Resources for exploring drug-target relationships. Nucleic Acids Res..

[34] Gilson M.K., Liu T., Baitaluk M., Nicola G., Hwang L., Chong J. (2016). BindingDB in 2015: A public database for medicinal chemistry, computational chemistry and systems pharmacology. Nucleic Acids Res..

[35] Landrum G. (2006). Others RDKit: Open-Source Cheminformatics. https://www.rdkit.org.

[36] Paszke A., Gross S., Massa F., Lerer A., Bradbury J., Chanan G., Killeen T., Lin Z., Gimelshein N., Antiga L. Pytorch: An imperative style, high-performance deep learning library. Proceedings of the Advances in Nneural Information Processing Systems.

[37] Fey M., Lenssen J.E. (2019). Fast graph representation learning with PyTorch Geometric. arXiv.

[38] Gönen M., Heller G. (2005). Concordance probability and discriminatory power in proportional hazards regression. Biometrika.

[39] Cichonska A., Pahikkala T., Szedmak S., Julkunen H., Airola A., Heinonen M., Aittokallio T., Rousu J. (2018). Learning with multiple pairwise kernels for drug bioactivity prediction. Bioinformatics.

[40] Smith T.F., Waterman M.S. (1981). Others Identification of common molecular subsequences. J. Mol. Biol..

[41] Kim S., Chen J., Cheng T., Gindulyte A., He J., He S., Li Q., Shoemaker B.A., Thiessen P.A., Yu B. (2019). PubChem 2019 update: Improved access to chemical data. Nucleic Acids Res..

[42] Zheng S., Li Y., Chen S., Xu J., Yang Y. (2020). Predicting drug-protein interaction using quasi-visual question answering system. Nat. Mach. Intell..

[43] Chen L., Tan X., Wang D., Zhong F., Liu X., Yang T., Luo X., Chen K., Jiang H., Zheng M. (2020). TransformerCPI: Improving compound--protein interaction prediction by sequence-based deep learning with self-attention mechanism and label reversal experiments. Bioinformatics.

